# Three-dimensional measurement of small inner surface profiles using feature-based 3-D panoramic registration

**DOI:** 10.1117/1.OE.56.1.014108

**Published:** 2017-01-30

**Authors:** Yuanzheng Gong, Eric J. Seibel

**Affiliations:** University of Washington, Mechanical Engineering Department, Seattle, Washington, United States

**Keywords:** three-dimensional surface reconstruction, three-dimensional measurement, point cloud registration, machine vision, optical metrology

## Abstract

Rapid development in the performance of sophisticated optical components, digital image sensors, and computer abilities along with decreasing costs has enabled three-dimensional (3-D) optical measurement to replace more traditional methods in manufacturing and quality control. The advantages of 3-D optical measurement, such as noncontact, high accuracy, rapid operation, and the ability for automation, are extremely valuable for inline manufacturing. However, most of the current optical approaches are eligible for exterior instead of internal surfaces of machined parts. A 3-D optical measurement approach is proposed based on machine vision for the 3-D profile measurement of tiny complex internal surfaces, such as internally threaded holes. To capture the full topographic extent (peak to valley) of threads, a side-view commercial rigid scope is used to collect images at known camera positions and orientations. A 3-D point cloud is generated with multiview stereo vision using linear motion of the test piece, which is repeated by a rotation to form additional point clouds. Registration of these point clouds into a complete reconstruction uses a proposed automated feature-based 3-D registration algorithm. The resulting 3-D reconstruction is compared with x-ray computed tomography to validate the feasibility of our proposed method for future robotically driven industrial 3-D inspection.

## 1 Introduction

Due to the rapid development of photonic sources, sensors, optomechatronics, and computer processing, the application of three-dimensional (3-D) optical measurement has been growing for inspection of machined external surfaces in manufacturing.^1^ With automation, 3-D optical measurement is increasingly replacing the traditionally used techniques in industry, which include mechanical gauging techniques such as the coordinate measurement machine (CMM),^2^ due to its characteristics of noncontact, nondestruction, high efficiency, and high accuracy.^3^ The generated 3-D point cloud can be used in quality control, inspection, virtual design, reverse engineering, simulation, and planning. Optical 3-D measurement techniques can generally be categorized as active and passive.^4^ The active approach obtains the 3-D coordinate data by interfering with the object using light, such as time-of-flight measurement,^5,6^ optical holography,^7^ optical to x-ray computed tomography (CT) scanning,^8^ and structured-light reconstruction.^9–12^ In contrast, passive 3-D systems require only a sensor, such as a camera, to collect digital images of the light scattering from the surface of an object. Typical techniques of passive vision contain stereo, structure from motion,^13^ and shape from shading.^14^

Commercial 3-D optical measurement systems are not adequate for providing high-quality reconstruction of small internal surfaces. For larger structures, the optical metrology equipment can fit inside and is becoming increasingly important in industry. An important application is crack inspection, quality control, installation, and even revamping of an existing pipeline in the oil/gas industry.^15^ In this case, the large diameter pipe can fit the complicated components of the active optical metrology instruments inside. For smaller structures, there is a need to rapidly and carefully inspect machined holes in engine manufacturing but a lack of optical 3-D inspection technologies that can physically fit inside the structures. An emerging application is highly accurate and reproducible 3-D inspection of tiny threaded holes.^16^ The main function of the threads is to hold different machined parts together, such as joining the engine block to the head in the automotive industry. Thus, the quality of the threads and their measured inner profiles are crucial for engine safety, performance, efficiency, and longevity.^16,17^ There are various sizes of internally threaded holes in the automotive engine block; most of them are smaller than 1 cm diameter. In this study, an internally threaded hole (M8) is used as the metal object to test our approach.

State-of-the-art techniques of optically measuring the small inner profile of internal structures have been developed more for inspection of internal human organs than for internal machined or fabricated structures. These endoscopic techniques range from conventional stereo^18^ to photometric stereo,^19,20^ structured light scope,^21,22^ time of flight,^23^ and 3-D shape from motion.^24^ In all cases, either the scopes are larger than 6-mm in diameter or the 3-D surface reconstruction is incomplete or at low resolution. Smaller optical scanning scopes based on low-coherence interferometry, called optical coherence tomography (OCT), were also developed for medical imaging.^25^ With OCT, to form 3-D surface profiles in tubes as small as 2 to 10 mm, a focused laser beam is scanned radially using a spinning mirror or prism.^26^ Although OCT was developed to image below the surface of translucent biological tissue, the technique can be applied to measuring opaque surface structures with lateral resolutions in the 10- to 30-µm range, but with limited depth of focus.^27^ With higher-speed 3-D imaging using Fourier domain OCT, new inline inspection applications are being developed for industry,^28^ although no 3-D surface reconstruction of threaded holes in shiny metal has been reported for these OCT techniques. Strong specular reflection of the shiny metal surface does lead to large artifacts in 3-D reconstructions.^29^ Moreover, OCT systems involve a complex (expensive) design of rapid probe rotation with high demands (e.g., robustness) that has been a barrier of entry into inline manufacturing systems.

Specifically for 3-D measurement of small pipes, Yoshizawa and Wakayama^30^ developed a 5-mm-diameter laser-scanning probe that strikes a cone mirror to generate a disk-like beam on the inner wall of the pipe. The reflected light is then captured by camera and processed with the triangulation principle to generate the 3-D profile. However, this device is still large in relation to the internally threaded blind hole in the engine block and could not accommodate the working range of 15 to 40 mm from the probe tip.^30^ In our previous study,^17^ an approach was proposed to measure the internal 3-D surfaces with an axial-stereo vision algorithm and a scanning fiber endoscope (SFE) of 1.2-mm outer diameter (OD).^31^ A dense point cloud was generated for a few threads from only two forward-view images along the hole axis. However, the accuracy limit was relatively large compared with the x-ray CT data that is used as the gold standard. In addition, the 80-deg field of view (FOV) of SFE could not be easily expanded to capture the thread profile from peak to valley to measure the inner (minor) and outer (major) diameters.

In this paper, we present a new 3-D optical measurement approach based on machine vision from a small caliper rigid scope to measure tiny complex internal surface profiles. This approach is formed by utilizing multiview stereo vision and a new proposed feature-based 3-D panoramic registration algorithm. The 3-D model of the entire internal surface (360 deg) is generated by registering a series of 3-D point clouds, which are reconstructed separately from side-view images of different perspectives with a multiview stereo algorithm, based on the robust two-dimensional (2-D) features and their 3-D correspondences. Details of this new approach and its theory will be presented in Sec. 2. In Sec. 3, we apply the general approach to a specific small test piece of standard-manufactured internally threaded M8 hole in aluminum using a commercial side-viewing rigid scope. The generated physical-scale 3-D point cloud is compared with the x-ray CT in Sec. 4. In the last section, the conclusion of our approach is drawn and the advantages of using a vision system for 3-D metrology are discussed, with future innovations proposed.

## 2 Methodology

### 2.1 Data Collection and Categorization

**Fig. 1:**
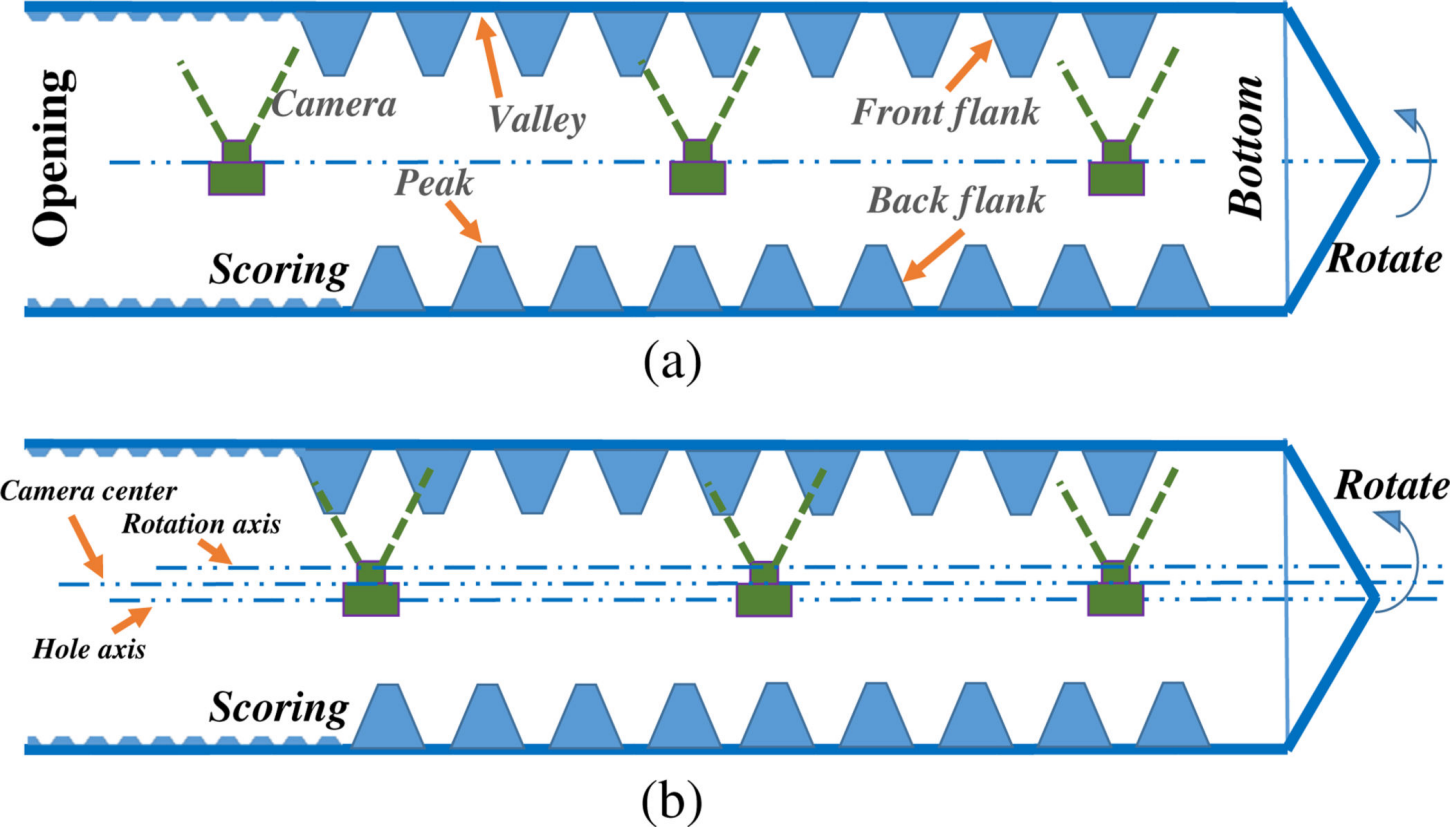
Diagram of a full axial scan of an internally threaded blind hole with 90-deg side-view camera.

In contrast with our previous approach of using axial images from a moving camera facing the hole bottom,^17^ a side-view camera is used in this study to capture the high topographic relief of the internal surface profile, such as the peaks and valleys of the internal threads that are labeled in Fig. 1(a). To ensure consistent and direct vision of the threads peak and valley, a high-resolution side-view camera is used but with limited FOV; see Fig. 1(a). To expand this limited FOV, we have to either rotate the camera or the object to guarantee that the entire 360 deg profile of the internal surface of the thread is captured.

The procedure of generating a scan of the entire hole containing a complex internal surface can be divided into four steps; see Fig 1(a): step (1) placing the camera at the opening of the blind hole, aligning with the hole axis, and facing to the side wall; step (2) taking a series of images as the camera moves inward along the hole axis with constant step distance; step (3) repositioning the camera at the hole opening and rotating it around the hole axis at a proper rotation degree to ensure image overlap with the images captured in step (2); step (4) repeating the steps (2) and (3) until finishing the entire scanning (360 deg) of the hole. For the image processing and 3-D reconstruction purpose, we have to make sure that each optical image contains more than 50% overlap within its sequential images,^32^ which we call “neighbor images.” In this study, the images that are collected with pure camera linear motion are called an “axial sequence,” and the ones that are captured with pure camera rotation are called an “angular sequence.” An axial expanse from the hole opening to the bottom within the same angular interval is captured in a “quadrant,” which is a set of axial sequential images, such as the image sequence that is collected in step (2). During inline manufacturing, the angular and axial information (camera position and orientation) can be retrieved from industrial robotic positioning systems. In this study, these camera extrinsic parameters (position and orientation) are considered prior. By taking advantage of the known camera pose information, the 3-D reconstruction of the threads is more accurate and efficient since it only needs to triangulate the 3-D point cloud representing the internal surface. There are two ways to achieve the relative rotational motion between the camera and the straight hole to complete 360 deg scanning: either rotate the camera within the fixed threaded hole or rotate the hole about the fixed camera. The choice depends on the particular objects to be measured, work space limitation, and implementation convenience.

Processing all of the images at once with state-of-art software VisualSfM,^13^ OpenMVG, or even the customized MATLAB^®^ code^24,33^ could not generate a satisfying 3-D reconstruction result. The reason is that in an ideal case these quadrants are captured with pure camera rotation (the camera baseline is zero), which would generate significant error when triangulating the depth and resulting gross reconstruction error.^34^ There are three axes in this setup: hole axis, rotation axis, and camera center trajectory line; see Fig. 1(b). In the ideal case, the complete 3-D model can be reconstructed by stacking each 3-D quadrant together since the three lines would be aligned; see Fig. 1(a), but this is impractical in current manufacturing.

Again referring to Fig. 1(b), the misalignment of the hole axis with the other two axes does not affect the reconstruction result, while the misalignment of the camera center line and rotation axis could generate geometrical discontinuity by stacking quadrants together directly. Typically, such misalignment is small and unknown, which could generate uncertain error in the 3-D surface reconstruction of the entire hole. To solve this practical problem, we propose reconstructing the 3-D point cloud of each quadrant from axial sequential images with a multiview stereo algorithm and registering (aligning) different quadrants point clouds using angular neighboring frames. This proposed data categorization and utilization can solve the high depth uncertainty problem caused by little camera baseline in vision-based 3-D reconstruction. To register two point clouds together, a feature-based 3-D registration algorithm was introduced in our previous work.^35^ Building on this work, a more advanced algorithm specific to the inspection of a threaded hole is proposed. This algorithm solves the 3-D registration problem of multiple point clouds that cover a 360 deg perspective of an object, which we call feature-based 3-D panoramic registration.

### 2.2 Feature-Based 3-D Panoramic Registration

Theoretically, the point clouds of different quadrants can be stacked together directly since the rotation angle of the camera is known from the robotic system. However, in practice, the camera center is not guaranteed to be perfectly aligned on the rotation axis; see Fig. 1(b). Without a known distance between camera center and rotation axis, direct stacking of multiple quadrants would result in a discontinuous surface. Iterative closest point (ICP)^36^ is a standard method for registering two point clouds together by iteratively revising the transformation to minimize the difference between them. However, ICP would fail in our case, resulting in the complete overlap of different quadrants due to their highly similar geometry.^35^ In this paper, a feature-based 3-D panoramic registration algorithm is proposed to achieve the registration of these highly similar quadrants from 360 deg scanning into an accurate 3-D surface reconstruction by taking advantage of the robustness of image features and their 3-D correspondences.

As mentioned above, each image has two kinds of neighbor images (axial and angular), which are used for 3-D reconstruction and registration purposes, respectively. Given a set of *N* axial images for the *i*’th quadrant *I_i_* = {*I*_*i*1_, *I*_*i*2_, …, *I_iN_*} that are captured by linear motion of the camera in one quadrant, feature detection and matching are applied to find the corresponding points in the image set. Let *F_i_* represent all the features that are detected from *I_i_*, and *G_i_* for the features used in the 3-D reconstruction. *G_i_* is a subset of *F_i_, G_i_* ∈ *F_i_*, since not every feature in an image has correspondences in its axial neighbors. With *a priori* knowledge of camera extrinsic parameters, a 3-D point cloud can be generated by the multiview stereo vision technique.^37^

To register two neighbor (*i*’th and *j*’th) quadrants together, we apply the same feature detection and matching algorithm on the angular neighbor pairs to find the corresponding features between these two quadrants. These correspondences are used for our proposed feature-based 3-D registration that are labeled as *H_i,j_* and *H_j,i_*, respectively. *H_i,j_* represents all the features in *F_i_* that have correspondences in *F_j_*, so *H_i,j_* ∈ *F_i_*. Here, for the common features that are used in both 3-D reconstruction and registration between the *i*’th and *j*’th quadrants, let *P_i,j_* represent their 3-D coordinates in the *i*’th quadrant coordinate system.

Considering the case of a fixed camera and a moving object, with * a priori* knowledge of the relative object rotation angle between two neighbor (*i*’th and *j*’th) quadrants, we have

(1) $$R_{i,j}(P_{i,j}-C_{i,j})=P_{j,i},$$

for each corresponding 3-D point, where *P_i,j_* and *P_j,i_* are known, *R_i,j_* is a 3 × 3 rotation matrix, and $C_{i,j}={\left[C_{i,j}^{x},C_{i,j}^{y},C_{i,j}^{z}\right]}^{T}$ is the linear offset of the two coordinate systems of different quadrants. Notice that since camera center axis and rotation axis are parallel, *C_i,j_* is a constant offset between the (*i*’th and *j*’th) quadrants. It is an overdetermined problem for the three variables *C_i,j_* in Eq. (1), since there are more than three correspondences between two neighbor quadrants with ≥50% image overlap. Considering that the rotation angles among neighbor quadrants are identical, *R* was used to replace *R_i,j_* in Eq. (1) for simplification. So the 3-D registration for each correspondence pair is

(2) $$\begin{array}{c} RP_{i,j}^{1}-P_{j,i}^{1}=RC_{i,j}\\ RP_{i,j}^{2}-P_{j,i}^{2}=RC_{i,j}\\ \vdots \\ RP_{i,j}^{m_{i,j}}-P_{j,i}^{m_{i,j}}=RC_{i,j} \end{array},$$

where *m_i,j_* is the total number of the correspondence pair in the *i*’th and *j*’th quadrants. By putting the left side of Eq. (2) into a column vector *b* with 3 × *m_i,j_* entries, and concatenating multiple *R* ’s vertically into *R̄*, we have *R̄_i,j_C_i,j_* = *b_i,j_*. Solving *C* for each pair of neighbor quadrants initially appears independent from other quadrants from this equation. However, we have to consider the scenario in this study of 360 deg scanning, which means the last quadrant shares image overlap with the first one. Suppose there are *k* quadrants in the scanning, we obtain

(3) $$\begin{array}{c} {\bar{R}}_{1,2}C_{1,2}=b_{1,2}\\ {\bar{R}}_{2,3}C_{2,3}=b_{2,3}\\ \vdots \\ {\bar{R}}_{k-1,k}C_{k-1,k}=b_{k-1,k}\\ {\bar{R}}_{k,1}C_{k,1}=b_{k,1}. \end{array}$$

Notice that *C*_*k*,1_ = −*C*_1,*k*_ = −(*C*_1,2_ + *C*_2, 3_ + … + *C*_*k*−1,*k*_); then, Eq. (3) can be written into matrix formas

(4) $$[\begin{array}{ccccc} {\bar{R}}_{1,2} & & & & \\ & {\bar{R}}_{2,3} & & & \\ & & \ddots & & \\ & & & & {\bar{R}}_{k-1,k}\\ {\bar{R}}_{k,1} & {\bar{R}}_{k,1} & {\bar{R}}_{k,1} & \ldots & {\bar{R}}_{k,1} \end{array}](\begin{array}{c} C_{1,2}\\ C_{2,3}\\ \vdots \\ C_{k-1,k} \end{array})=(\begin{array}{c} b_{1,2}\\ b_{2,3}\\ \vdots \\ b_{k-1,k}\\ -b_{k,1} \end{array}).$$

Let *R̂* represent the coefficient matrix in Eq. (4) and *b̂* the vector of constant terms, resulting in

(5) $$(\begin{array}{c} C_{1,2}\\ C_{2,3}\\ \vdots \\ C_{k-1,k} \end{array})={\left({\hat{R}}^{T}\hat{R}\right)}^{-1}{\hat{R}}^{T}\hat{b}.$$

Since *R* is a full rank rotation matrix, the concatenating matrix *R̄* is full rank too. Thus, *R̂* has full rank, then (*R̂^T^R̂*)^−1^ exists. The calculation of this inverse matrix is very efficient since *R̂^T^R̂* is a small square matrix with a side of 3(*k* − 1). For instance, the quadrant number *k* = 12 in our study as the camera FOV is about 60 deg. By solving *C*_1, 2_, *C*_2, 3_*, … *C*_*k*−1, *k*_* from Eq. (5), all the quadrants can be registered together to generate the entire hole inner surface after transforming to the same coordinate system.

## 3 Experiment

### 3.1 Experiment Setup

**Fig. 2:**
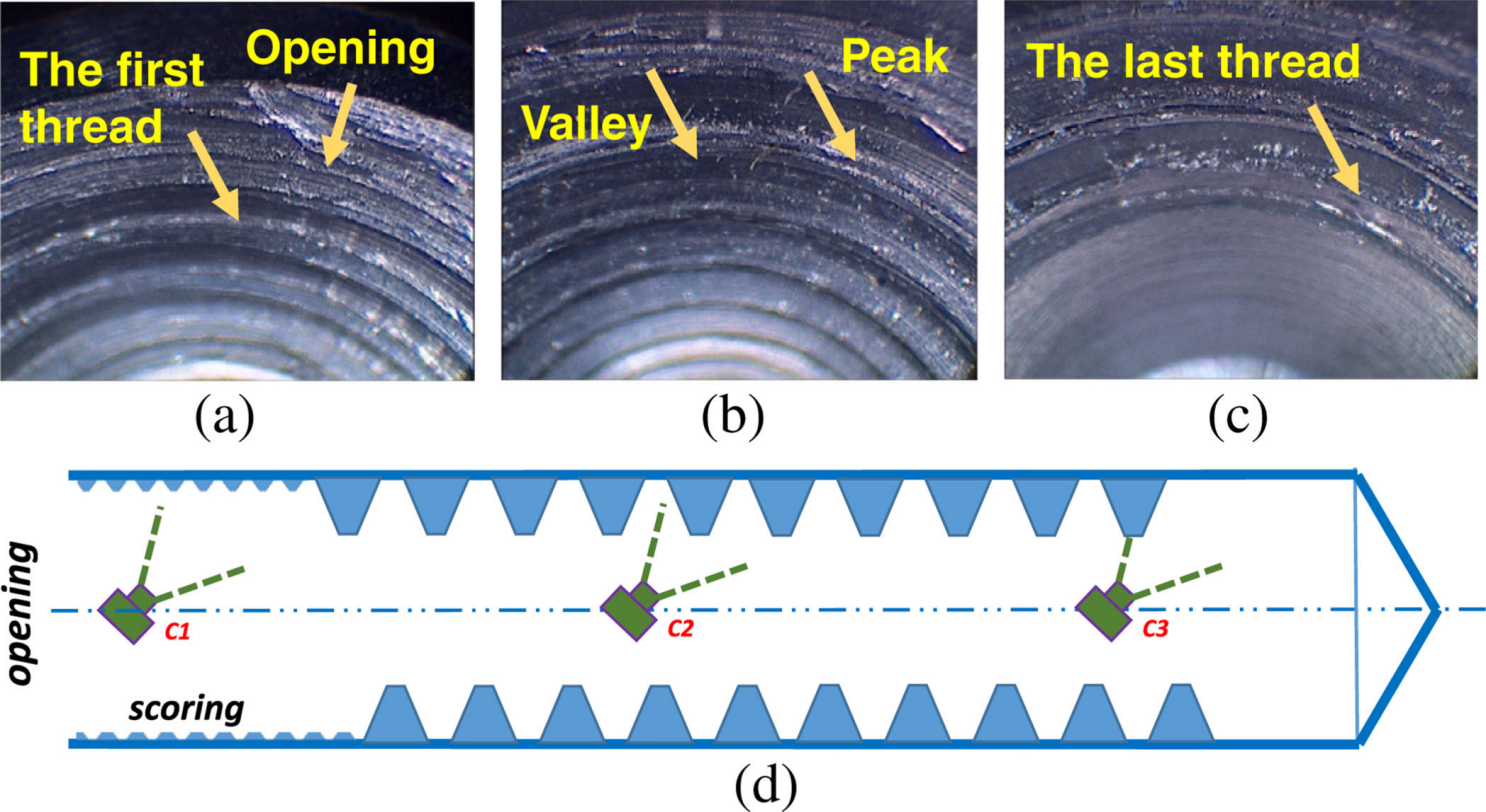
The experiment setup of 3-D optical measurement of M8 threaded blind hole with commercial borescope. (a) The test piece with recessed coarse M8 threads machined in shiny aluminum metal; (b) the distal end of a rigid scope of 45-deg side-view angle and with camera resolution of 1280 × 1024 pixel; (c) the camera processor and illumination source of the commercial rigid scope; and (d) the experiment setup mounted on an optical breadboard with components of micropositioning linear and rotation stages, test piece, scope, and customized scope holders.

To demonstrate the feasibility of our proposed new approach to the 3-D optical measurement of a machined internal structure with a tiny internal surface, an experiment was performed to generate the 3-D point cloud from within a shiny metal object and compared with current measurement techniques. In this study, an internally threaded blind hole with a <7 mm minor diameter (M8) was chosen as the test piece (object); see Fig. 2(a). A relatively large rigid scope of 5.5-mm OD (Stryker™ scope model #502–503–045) was chosen for the ease of a stable setup with high-resolution side-viewing capability; see Figs. 2(b)–2(d). Due to the limitation of this commercial scope and external camera system, it only can achieve 45-deg side-view angle with 60 deg FOV, 1280 × 1024 pixels resolution, and 40-cm working length. Although smaller rigid and flexible scopes with forward viewing were available, there was insufficient time to create fixtures using a right-angle mirror at the distal tip that fit within the small size of this M8 hole while preserving the FOV of 60 deg.

To ensure stability, precision, and accuracy, an optical rail was fixed on an optical breadboard, on which three identical kits of optomechanical components (rail carrier, post, and holder) were set up to hold the rigid scope horizontally; see Fig. 2(d). Instead of rotating and translating the scope that connected to a bulky illumination system, the scope remained stationary during the entire experimental procedure. To achieve relative linear translation and rotation for the full axial and 360 deg scanning, the test piece was attached on a high-quality rotation platform, which was mounted on a micropositioning stage (ULTRAlign™ precision integrated crossed-roller bearing linear stage, model 462-xyz-m); see Fig. 2(d). The reason for using micropositioning linear and rotation stages is to get the accurate relative position and angle data between the test piece and scope, which can be retrieved from modern industrial robotic systems in future industrial applications.

**Fig. 3:**
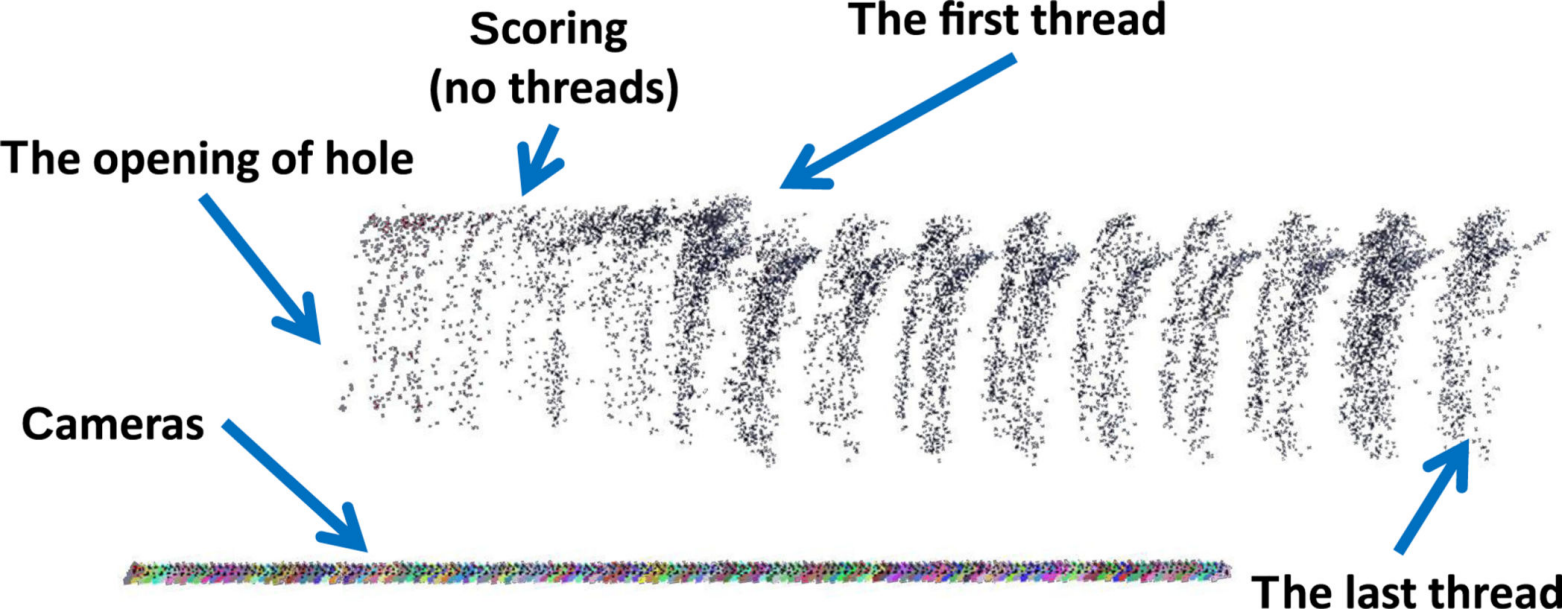
Three endoscopic image examples of the internally threaded blind M8 hole from the rigid scope with a 45-deg side-viewing angle. (a) The first frame in a quadrant with opening of the hole and the first thread labeled; (b) the thread peak and valley, shown as bright band and dark area, respectively; (c) the last frame of a quadrant containing the last thread and the bottom of the blind hole; (d) the diagram showing the camera positions where (a)–(c) were captured, listed as *C*1, *C*2, and *C*3, respectively.

The image collection starts with placing the scope at the center of the opening of the threaded hole and pointing inward to the hole bottom, after camera calibration with open toolbox in OpenCV. To guarantee 50% image overlap among the sequential images, the test piece was moved forward by micropositioning stage with a known step distance of 0.1 mm in this experiment. One image was collected by the rigid scope system at each step; see Fig. 2(c). A sequence of axial images was recorded, which covers about 60 deg of the threaded hole from hole opening to the bottom. Figure 3 shows endoscopic images of the internal threads, with clear vision of the peak (curved bright belt) and the valley (dark area just axially beyond the peak). The similar peak-valley pattern is repeated in the sequence from Figs. 3(a) to 3(c), as shown in Fig. 3(d). The effect of specular reflections is insignificant due to the light source design of the scope and the lack of consistency in the sequence of images.

After scanning one quadrant, the test piece was repositioned, so the scope was at the center of the opening. For ≥50% image overlap between neighbor quadrants, the test piece was rotated 30 deg using the rotation stage on which the scope was mounted. The same procedure above was then performed to collect a set of axial images for the second quadrant. By repeating this, 12 sets of sequence images were recorded for the 360 deg scanning of the entire hole.

### 3.2 3-D Reconstruction with Feature-Based Panoramic Registration

**Fig. 4:**
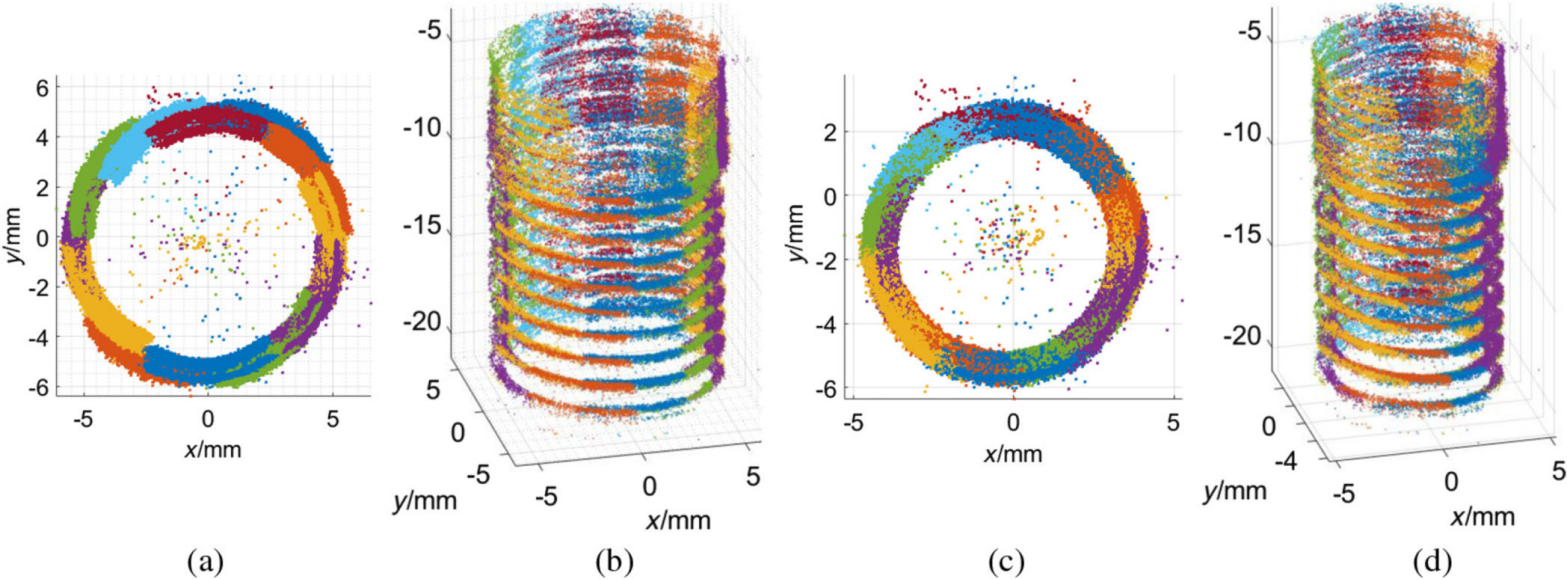
The 3-D reconstruction of one quadrant of the recessed internally threaded hole from a sequence of axial images with known camera position and orientation.

In this experiment, 155 axial images were captured for each quadrant covering the range from the opening through the bottom. The scale-invariant feature transform (SIFT) algorithm^38,39^ was applied to find robust feature points from each image. Random sample consensus was used to select the robust matching feature pairs among all the axial images. Since the relative camera poses (position and orientation) were known from the micropositioning linear and rotation stage, multiview stereo vision was performed to generate a sparse 3-D point cloud of each quadrant. Here, we utilized state-of-art software VisualSfM^13^ for the processing and visualization for each quadrant, shown in Fig. 4, with labels of the opening of threaded hole and the start and end of the threads. The colorful straight line in Fig. 4 is the series of camera positions of the line of these axial images as the micropositioning stage moved inward.

**Fig. 5:**
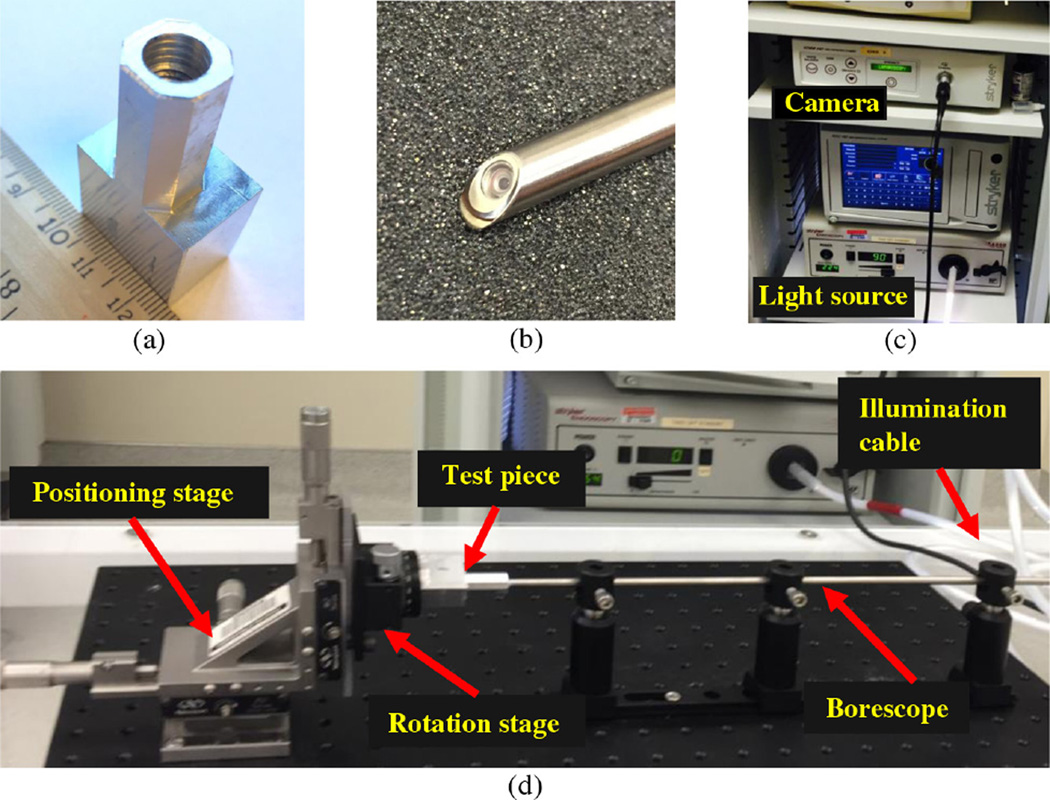
The 3-D reconstruction result of entire scored (upper) and threaded portion of a blind hole with and without feature-based 3-D panoramic registration algorithm. (a, b) The top and side perspective views of the reconstructed model by direct stacking without registration, respectively; (c, d) the top and side perspective views of improved 3-D reconstruction with feature-based registration, respectively.

Repeating the above process, the point clouds of 12 quadrants, which contain different total numbers of 3-D points, were generated separately. In our experiment, there were about 12,000 points generated to represent each quadrant. In the case that the rotation axis aligned with the camera centers perfectly, all the quadrants can be directly stacked together after pure rotation. However, maintaining this accurate alignment is impractical for in-line manufacturing. Trying to achieve accurate alignment manually in our setup results in the direct stack of multiple quadrants, labeled in different colors in Figs. 5(a) and 5(b).

To register all quadrants together, the standard approach using ICP did not work well for this case due to the highly similar geometry of these quadrants.^35^ Our proposed feature-based 3-D panoramic registration was applied to take advantage of the high accuracy, robustness, and distinguish-ability of SIFT features. By applying the same feature detection and matching algorithm on the angular image sequences of two neighbor quadrants (*i*’th and *j*’th), the common features (*P_i,j_* and *P_j,i_*) were detected. Their 3-D coordinates can be easily retrieved from the reconstructed quadrant models. 3-D registration of these two quadrants can be achieved by calculating the rigid transformation matrix between *P_i,j_* and *P_j,i_*. With known rotation angle, the calculation was simplified as shown in Eq. (5) to solve the misalignment problem between the rotation axis and camera centers; see Fig. 1. The final registration is an improvement over the direct stacking method, as shown in Figs. 5(c) and 5(d) with the top and front views.

**Fig. 6:**
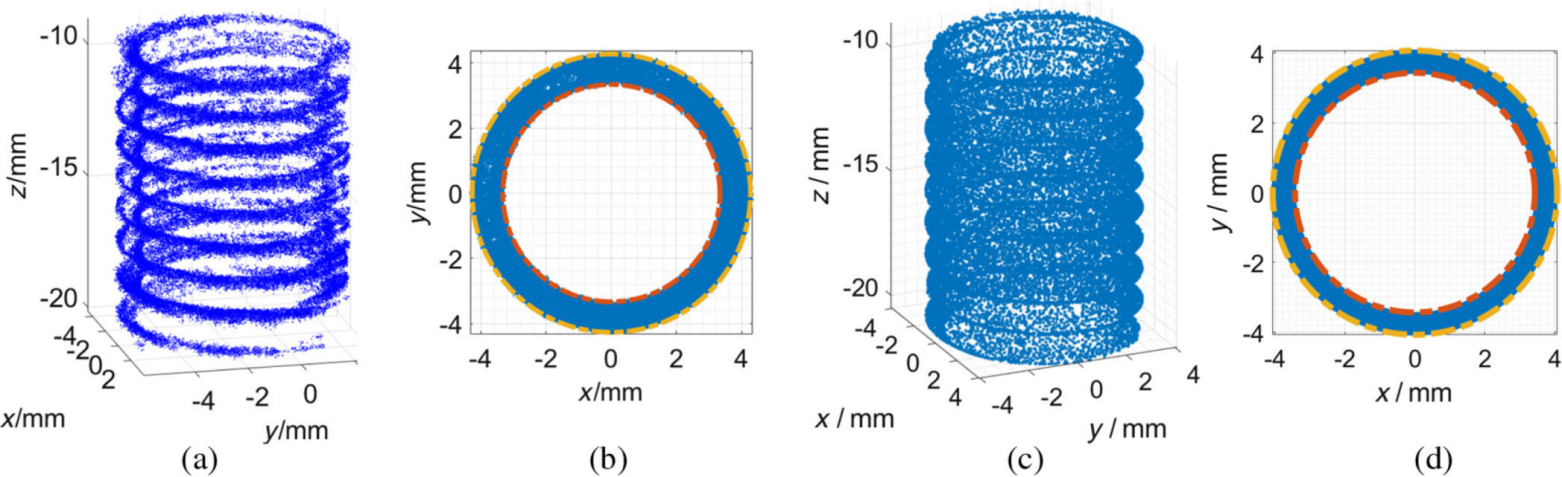
The comparison of x-ray CT and our measurement method on minor and major diameters based on 3-D point clouds of internal threads. (a) Our generated 3-D point cloud after statistical filter; (b) the measurement of minor (red) and major (yellow) diameters by calculating the smallest and largest distances from the projected 2-D points to the center; (c) the x-ray data of the same internal threads with front and back flanks; (d) the measurement of minor (red) and major (yellow) diameters by calculating the smallest and largest distances from the projected x-ray data to the center.

Figure 5(c) shows that there were a few outliers existing in the final registered 3-D model, which might be caused by incorrect matching feature pairs. In this study, a statistical filter,^40^ which is based on the spatial distribution of each point, was applied to improve the quality of the reconstructed model by identifying and trimming these outliers. Specifically, a mean distance for each point to its 30 nearest neighbors was computed in our study, with the assumption that the mean distances for all the points exhibit a Gaussian distribution with a mean and standard deviation.^41^ The points whose mean distances are 3× standard deviation (3σ) larger than average were considered outliers^42^ and removed from the final model, as shown in Figs. 6(a) and 6(b).

All the software programs were run on a notebook Dell Precision M4700 with 20.0-GB memory and 2.7-GHz Intel i7-3740QM CPUs in a 64-bit Windows operating system. The 3-D reconstruction of each quadrant took about half an hour in C++; the 3-D registration required about 24 min to generate the entire hole; and the noise reduction by statistical filter took 1.4 s to clean the outliers with an open source of Point Cloud Library.

## 4 Results

With a 45-deg side-view camera, the back flank of each thread was not fully visible and not recorded. Therefore, the reconstruction of the entire hole only showed a representative model of the front flank of the threads; see Fig. 6(a). This lack of data may not be critical. To validate the feasibility of our approach to be a potential in-line measurement tool, several important thread parameters (minor diameter, major diameter, and pitch) must be measured in the virtual 3-D space and then compared with the standard tactile measurement CMM and noncontact x-ray CT. Minor diameter is defined as the minimum diameter of the threads and major diameter is the largest one; pitch is the axial distance between two neighbor threads. In this data analysis, the M8 coarse hole parameters were listed in Table 1 as a reference.

To measure the minor and major diameters from the 3-D point cloud, only the internal threads beyond the scored bore opening are utilized for the following data analysis and comparison. Our reconstructed threads are projected to a polar plane that is perpendicular to the axis of the hole; see Fig. 6. The center of this projected 2-D point set is calculated by fitting a circle. The minor radius of the hole was the smallest distance from the center to each projected point, labeled in orange; and the major radius corresponds to the largest distance, labeled in yellow; see Fig. 6(b). The same analysis was performed on the x-ray CT data, with the result shown in Fig. 6(d).

In the data acquisition procedure with x-ray CT, the test piece is positioned on a precision rotational stage. The x-ray light source generates a conic beam of electron that penetrates the test piece and is collected by a 2-D detector as a digital radiograph image. During the rotation of the test piece at a constant step, a sequence of radiograph images is collected. In our study, the x-ray data collection is performed by a recently installed and calibrated X5000 CT scanning system (North Star Imaging Inc.). The 3-D point cloud is generated by a commercial software efX-ct with its default iso value. Iso value is a number between 0 and 1 that represents the intersection point where the surface will be generated based on density values within the volume. A surface is defined as a transition between two densities of materials.

Table 1 lists the measurement results of minor diameter, major diameter, and pitch with x-ray CT and our method compared to the M8 specifications.^43^ All measurements of minor diameter were within the M8-specified range. Our method generated a −2.9% difference against the x-ray CT. For the major diameter, the measurement result of our method generates +4.8% against with x-ray data. The measurement results of pitch of x-ray CT and our method are consistent with the manufacture standard of 1.25 mm for an M8 threaded hole, with an error of −0.001 mm (−0.08%) and +0.005 mm (+0.4%), respectively.

Additional analysis was performed to compare the measurement results of our method and x-ray CT. In this study, there were nine threads in the threaded hole; see Fig. 6. The minor and major diameters of each thread of the threaded hole were measured by the same method discussed above. The average and standard deviation values for each 3-D models were calculated and listed in Table 2. There is −2.7% and +4.6% difference on the average value of the measurements of minor and major diameters, respectively. The difference of standard deviation is only −2.6% and +1.3% for minor and major diameters, respectively.

As mentioned above, the iso value is the threshold to distinguish material and nonmaterial within the volume. In our case, a lower iso value may capture surrounding air as the metal threads; a higher iso excludes materials within the threads. Therefore, different iso values generate various measurement results of minor and major diameters. Table 3 lists the comparison of the measurement results with different iso values. We can see that a lower iso creates smaller minor and major diameters; while a larger iso value “erodes” the material and generates larger diameters. Choosing different iso values (2 and 8) generates 7.2% and 2.1% difference in the measurement of minor and major diameters, respectively. In contrast, our method, in this case, generates less than 0.1% difference in the diameter measurements with 2.5σ and 3.5σ statistical filters.

Further analysis was performed between our method and x-ray CT for the similarity comparison of two point clouds. X-ray CT generated a dense 3-D point cloud of the whole test piece including the external surface, since radiation x-ray can penetrate the entire object. The 3-D point density of our reconstruction result varies across regions of thread peak, flank, and valley; see Fig. 6(a). This is because our optical reconstruction is based on 2-D feature points, which distribute unevenly on the endoscopic images. More specifically, there is a higher density of SIFT features on the peaks than valleys with a lack of features on the visible flanks observed at a 45-deg side-viewing perspective. In contrast, the obtained x-ray point cloud had a more uniform 3-D distribution of points on the threads’ surfaces of peak, valley, and flank; see Fig. 6(c).

## 5 Discussion and Conclusion

This paper proposed a new approach for the 3-D optical measurement of small holes with complex and repeating side-wall geometries using a machine vision technique and feature-based 3-D panoramic registration. Due to the size limitation of the small object, the traditional measurement methods do not work well,^2,16,30,44^ except x-ray CT. However, the x-ray CT approach is infeasible for in-line measuring of small threaded holes in thick metal blocks. As mentioned above, rotation and linear motions are performed on the test piece; the x-ray CT scanning process takes about 1 h for a complete and fine scan of our small test piece. It will take much more time for thick engine blocks, so it is not feasible for practical applications. Moreover, the x-ray CT system took about 20 min for the image processing to generate a single 3-D model with a super computer (256 G RAM memory, 24 cores of Intel Xeon CPUs, and 4 NVidia Quadro M6000).

In our approach, a side-view medical scope was utilized to capture the high topographic relief of the internal surface profiles of the machined spiral thread. However, this required a complete rotation of the camera view within the blind hole; it took over an hour to acquire the data manually. This process can be sped up by robotically driving the camera within the threaded hole. The hours required for image processing can be dramatically reduced using high-speed and efficient computing, as is done for the x-ray CT system. Considering the practical application with the misalignment issue of the rotation axis and camera center, a feature-based 3-D panoramic registration algorithm was proposed in this paper and applied to align multiple quadrants of 360-deg perspective together by taking advantage of 2-D and 3-D information of common features among different quadrants. As the gold standard of point cloud registration, ICP could not be used to build the multiple quadrants into a complete model due to the repetitive geometrical nature of the spiral thread.^35^ The existing outliers in the registered model were then trimmed by a statistical filter based on their spatial distribution. The comparison among the final reconstructed point cloud and x-ray CT data shows that x-ray CT generates a much denser point cloud than our vision-based 3-D reconstruction approach, but it also comes with a parameter (iso) that greatly affects the measurement. The comparison also validated the feasibility of the proposed approach for being a potential method for the 3-D optical in-line measurement for these small internal surface profiles in metal.

Although a 45-deg side-view scope was used in the case study experiment, any wide-angle side-viewing scope could be used as long as the valley was clearly resolved with image-based features to measure the major diameter. By analyzing images acquired with a right-angled mirror at the distal end of a forward viewing scope, such as a rigid commercial scope or the smaller flexible SFE, this 90-deg side-view appears to generate a more unbiased (centered) distribution of image features at the bottom of the valleys, where both flanks are visible. In the experiment, 0.1-mm step distance was chosen as the camera moves inward to maintain ≥50% image overlap. Different sizes of the step can be utilized depending on the camera orientation, FOV, and size of hole. With a larger camera FOVor larger hole, a larger step distance can satisfy the image overlap requirement, resulting in fewer images that need to be collected and processed. This refinement would dramatically decrease the computation time in both reconstruction and registration with an algorithm complexity of *O* (*n*^2^).

In the practical application of quality control of the internal threads, go/no-go gauges are used as the gold standard to judge “yes/no” for each dimensional parameter of the threaded hole. This binary decision can be automated to perform rapidly but with a major flaw from a data perspective: each parameter can only be defined to be within lower and upper bounds (go and no-go) instead of a specific value. The definition of the bounds highly depends on the interval of the gauge size. With the 3-D point cloud that is generated by our approach (or x-ray CT), future work is to measure these parameters in the virtual space by a virtual gauge. The generated 3-D point cloud can provide graded parameter values instead of a range. Moreover, point defects such as pores, chips, and cracks on the internal thread surface can be detected from the dense point cloud, which cannot be achieved by go/no-go gauging. The advantages of using our approach to currently used metrology: (1) ability of noncontact inspection; (2) accessibility of variable data; (3) independence from operators’ skills; (4) high automatability with robotics; (5) reduced labor and maintenance cost; (6) potential high efficiency with high-power computer; (7) feasibility of in-line measurement; and (8) vision of the threaded surface for point, line (scratch), and spectroscopic (color) inspection. Direct vision is a big advantage of our method compared with other technologies that are currently feasible for the 3-D measurement of small internal surfaces: x-ray CT,^8^ OCT,^27,28^ and laser ring beam scanning.^30^ Duplicating human vision has a long-term major advantage because it can rapidly harness the incredible capabilities being generated in computer vision and machine learning using deep neural networks for artificial intelligence analyses.^45^ Moreover, vision provides an efficient and robust feature-based tool for registration and comparison among multiple 3-D reconstructions.

Future work on advancing our approach will solve several limitations listed below. (1) During the image collection, accurate camera position and rotation are demanded for the 3-D reconstruction of each quadrant. By applying a bound constrained bundle adjustment (BCBA) algorithm^46^ with prior knowledge of the accuracy and precision of industrial robot behavior using a precalibrated inspection scope, our approach can be improved to handle the real-world scenario of inaccurate camera poses. BCBA can also be used to solve the registration problem if the relative rotation angles among different quadrants are inaccurate or even unknown. (2) The image collection of multiple quadrants requires multiple times for the camera to move inward and outward, which increases the time and complexity in practical applications. This may be solved by utilizing a cone mirror^47^ placed in front of a forward-view camera. At each axial position, one frame is captured with a side-view on the 360-deg wall of the hole. Another advantage of this design is that it avoids the 3-D registration of multiple quadrants, which may result in more accurate and efficient 3-D surface model formation. The drawbacks are that a new feature detection algorithm is required for axial images and a more complex calibration method is needed for camera/mirror setup. (3) Higher density point clouds are needed, especially at the surface extremes (peaks and valleys) used for dimensional measurements in this application. The state-of-the-art dense multiview stereo technique may dramatically increase the density of the final point cloud with *s-t* cut optimization and with visibility and photoconsistency constraints.^48^ (4) The current design only works for straight hole, different test pieces; an imaging sensor will be utilized in future work for the 3-D reconstruction of curvature tubes. This application can be used for medical purpose, such as the quality check of implanted stents.

## Figures and Tables

**Table 1:** Measurements of minor diameter, major diameter, and pitch of the threaded M8 hole with x-ray CT and our method compared to the thread specification.

	Minor diameter (mm)	Major diameter (mm)	Pitch (mm)
X-ray CT with default iso	6.866	8.145	1.249
Our method with 3σ filter	6.667	8.539	1.255
M8 coarse specification	6.647 to 6.912	8.000 to 8.340	1.250

**Table 2:** Measurements of minor and major diameters of each thread in the generated 3-D models of the x-ray CT and our method.

	Minor diameter		Major diameter	
		
	Average (mm)	Std (mm)	Average (mm)	Std (mm)
X-ray CT with default iso	6.876	0.1131	8.127	0.0152
Our method with 3σ filter	6.690	0.1099	8.524	0.0154

**Table 3:** Comparison of the x-ray CT measurement results with different iso values.

Iso value	Minor diameter (mm)	Major diameter (mm)
2	6.467	8.036
4.83 (default)	6.866	8.145
4	6.850	8.128
8	6.933	8.207
